# Assessment of the Impact of Zoledronic Acid on Ovariectomized Osteoporosis Model Using Micro-CT Scanning

**DOI:** 10.1371/journal.pone.0132104

**Published:** 2015-07-06

**Authors:** Bo Shuai, Lin Shen, Yanping Yang, Chen Ma, Rui Zhu, Xiaojuan Xu

**Affiliations:** Department of Integrated Traditional Chinese and Western Medicine, Union Hospital, Tongji Medical College, Huazhong University of Science and Technology, Wuhan, 430022, China; Nanjing Medical University, CHINA

## Abstract

**Purpose/Objective:**

Prompted by preliminary findings, this study was conducted to investigate the impact of zoledronic acid on the cancellous bone microstructure and its effect on the level of β-catenin in a mouse model of postmenopausal osteoporosis.

**Methods and Materials:**

96 8-week-old specific-pathogen-free C57BL/6 mice were randomly divided into 4 groups (24 per group): a sham group, an ovariectomized osteoporosis model group, an estradiol-treated group, and a zoledronic acid-treated group. Five months after surgery, the third lumbar vertebra and left femur of the animals were dissected and scanned using micro-computed tomography (micro-CT) to acquire three-dimensional imagery of their cancellous bone microstructure. The impact of ovariectomy, the effect of estradiol, and the effect of zoledronic acid intervention on cancellous bone microstructure, as well as on the expression of β-catenin, were evaluated.

**Results:**

The estradiol-treated and the zoledronic acid-treated group exhibited a significant increase in the bone volume fraction, trabecular number, trabecular thickness, bone surface to bone volume ratio (BS/BV), and β-catenin expression, when compared with those of the control group (*P* <0.01). In contrast, the structure model index, trabecular separation, and BS/BV were significantly lower compared with those of the model group (*P* <0.01). No differences were observed in the above parameters between animals of the zoledronic acid-treated and the estradiol-treated group.

**Conclusion:**

These results suggest that increased β-catenin expression may be the mechanism underlying zoledronic acid-related improvement in the cancellous bone microstructure in ovariectomized mice. Our findings provide a scientific rationale for using zoledronic acid as a therapeutic intervention to prevent bone loss in post-menopausal women.

## Introduction

Osteoporosis has often been evaluated by measuring the mass of mineral per unit volume of bone, or bone mineral density (BMD). Various methods for BMD measurement have been developed, including X-ray measurements, radiographic absorptiometry (RA), single-photon absorptiometry (SPA), dual-photon absorptiometry (DPA), single-energy X-ray absorptiometry (SEXA), and dual-energy X-ray absorptiometry (DEXA) [1]. The accuracy of these measurements has gradually improved. Using BMD as an indicator for the evaluation and diagnosis of osteoporosis has made a significant contribution to our understanding of the pathogenesis of osteoporosis, as well as to its prevention, treatment, monitoring, and prognosis. However, BMD measurement alone does not provide us with all the data we need, because it is only a quantitative measurement of bone mineral and cannot tell us about the stability or mechanical strength of cancellous bone [2–5].

The pathological changes caused by osteoporosis occur mainly in cancellous bone, a structure of interconnected rods and plates that form a three-dimensional branching lattice [6]. Typically, osteoporosis causes the amount of trabecular bone to be reduced and the bone to become thinner, while the intertrabecular space enlarges and the interconnected structure of trabecular bone is disrupted [7]. Trabecular perforation occurs in its plate-like structures, and normal plate-like trabeculae may be converted into thinner rod-like structures which can even disappear altogether, thereby increasing the risk of fracture [8]. The assessment of microstructural changes in cancellous bone is a key element in the study of osteoporotic fracture and the evaluation of anti-osteoporotic agents [7]. Micro-computed tomography (micro-CT), a high-resolution scanning technique, is considered the most sensitive and accurate method for the study of osteoporosis caused by various conditions and for the evaluation of disease severity. Micro-CT allows 3-dimension (3-D) analysis of bone density and microstructure and allows us to assess the structure and functionality of the bone through assessment of the trabecular structure [9–11].

β-Catenin, an important signaling molecule of the Wnt pathway, plays a major role in osteoblast differentiation, proliferation, and apoptosis [12]. It has been reported that estrogen regulation of β-catenin-dependent transcription might also occur independently of canonical Wnt signaling [13–15]. It has also been reported that p38 MAPK-regulated GSK-3β/β-Catenin-Bcl-xL signaling is required for cells’ survival of zoledronic acid-induced apoptosis in both osteoclast precursors and osteoclasts, and that the pathway enhanced zoledronic acid’s effect on increasing the bone mineral density of ovariectomized mice [16]. Zoledronic acid is commonly used in the treatment of osteoporosis, particularly in post-menopausal women, but its mechanism of action remains to be elucidated, in particular its link to β-catenin expression.

In the present study, the micro-CT technique was employed to reconstruct and analyze the 3-D structure of cancellous bone in the lumbar vertebra and the femur of an ovariectomy-induced osteoporosis mouse model to evaluate the effect of zoledronic acid on osteoporosis. Furthermore, the underlying mechanism of the effect of zoledronic acid was investigated through the direct assessment of the shape, structure, and 3-D bone geometry of the trabeculae.

## Materials and Methods

### Animal treatment and grouping

96 8-week-old specific-pathogen-free (SPF) C57BL/6 mice, weighing 22g to 26g, were purchased from and maintained at the Experimental Animal Center of Huazhong University of Science and Technology (HUST), Wuhan, China (certificate: NO. 0237269; Hubei provincial experimental animal facility permit: SYXK [Hubei] 2010–0057). Disinfected food and water were provided under sterile conditions. After 1 month of normal feeding, the animals were randomly divided into 4 groups consisting of 24 animals each: the sham, ovariectomized osteoporosis model, estradiol-treated, and zoledronic acid-treated groups. This study was carried out in strict accordance with the recommendations in the Guide for the Care and Use of Laboratory Animals of the Huazhong University of Science and Technology. The protocol was approved by the Committee on the Ethics of Animal Experiments of the Huazhong University of Science and Technology. All surgery was performed under sodium pentobarbital anesthesia, and all efforts were made to minimize suffering.

### Ovariectomized osteoporosis model group

The animals in the ovariectomized osteoporosis model group were anaesthetized with an intraperitoneal injection of 1% pentobarbital sodium (30mg/kg b.w.) and maintained in prone position. Ovariectomy was performed via a midline dorsal incision under sterile conditions after which both ovaries were identified adjacent to the inferior pole of the kidneys in the peritoneal cavity. The blood vessels were tied off using a no. 4 suture, and both ovaries were removed. The tissue layers were closed and sutured. Starting from post-operative day 5, a pap smear was taken from each animal once each day for 5 consecutive days. A successful bilateral ovariectomy was confirmed by a pap smear screening showing the absence of keratosis. Samples exhibiting keratosis were discarded. The animals received 0.5ml saline/day via intragastric gavage from post-operative day 7 onwards.

### Sham group

The animals in the sham group were anaesthetized with an intraperitoneal injection of 1% pentobarbital sodium (30mg/kg b.w.) and maintained in prone position. Ovariectomy was performed via a midline dorsal incision under sterile conditions after which both ovaries were identified adjacent to the inferior pole of the kidneys in the peritoneal cavity. Only the adipose tissue surrounding the ovaries, with a weight equivalent to that of both ovaries, was removed. The tissue layers were closed and sutured and the animals received 0.5ml saline/day via intragastric gavage from post-operative day 7 onwards.

### Estradiol-treated group

The animals in the estradiol-treated group underwent the same bilateral ovariectomy procedures as those in the ovariectomized osteoporosis model group did and were fed 0.5ml of a solution containing dissolved estradiol valerate tablets (Bu Jia Le) [17] from post-operative day 7 onwards.

### Zoledronic acid treated group

The animals in the zoledronic acid-treated group underwent the same bilateral ovariectomy procedures as those in the ovariectomized osteoporosis model group and were fed 0.5ml of zoledronic acid from post-operative day 7 onwards according to a previously described method [18]. The doses of zoledronic acid were calculated using data from a dose-response study previously conducted in a rat osteoporosis model [12].

None of the animals received antibiotics after surgery. Animals were fed under consistent conditions and movement was allowed *ad libitum*. After a 3-month treatment period, the animals were sacrificed using an overdose of anesthesia and the 3rd lumbar vertebra and the left femur of the animals were surgically removed and dissected immediately. The attached muscle and soft tissue were also removed and the samples were wrapped in saline-soaked gauze for further tests.

### Immunohistochemical study of β-catenin expression in bone tissue

4μm bone tissue sections were deparaffinized with xylene twice for 10min, followed by rehydration in decreasing concentrations of ethanol (100%, 90%, and 80%, respectively) for 10min per concentration. After a 5-min wash with phosphate-buffered saline (PBS), the samples were incubated in 3% methanol-hydrogen peroxide at room temperature for 15min to deactivate endogenous peroxidase. Samples were then washed 3 times with PBS (5min each), treated with a heated ethylene diamine tetraacetic acid antigen retrieval solution in a microwave oven set at high heat mode for 2.5min and on defrost mode for 10min, and were cooled for 30min to room temperature. Samples were washed 3 times with PBS (5min per wash), blocked with 10% goat serum, and incubated in a wet box at room temperature for 30min. This was followed by incubation with a goat anti-mouse β-catenin antibody in a wet box at room temperature for 30min. Following 3 washes with PBS (5min per wash), the samples were incubated with 3,3’-diaminobenzidine (DAB) and the chromogenic reaction was stopped by washing with tap water. The samples were stained with Mayer’s hematoxylin for 20sec and rinsed in tap water. Finally, the slides were dehydrated in a graded ethanol series (80%, 90%, and 100%) for 10min per concentration, cleared in xylene, and mounted in neutral resin. The immunostaining was assessed by two independent observers. The percentage of immunostaining was evaluated, and the staining intensity was recorded.

### Micro-CT imaging

The trabecular microstructure and a 3-D image of the 3rd lumbar vertebra and left femur were scanned by micro-CT (μCT20; Scanco Medical, Bassersdorf, Switerland, provided by the Hospital of Stomatology, Wuhan University, Wuhan, China). Details of this micro-CT scanner have been described previously [8]. In short, samples were placed in the sample holder of the scanner and scans were made along the longitudinal axis of the specimen. Consecutive micro-CT images were captured. The image size was set at 1024×1024 pixels. Scans were made using the following scanning parameters: 90kV, 88μA, a 2° rotation step, a 360° rotation angle, a total of 100 slices (lumbar vertebral) and 250 slices (left femur) at 14μm slice thickness. Image reconstruction was implemented using VMS system in cluster configuration with a voxel size of 14μm×14μm×14μm. To obtain the original 3D image of the 3rd lumbar vertebral and left femur, a threshold value of 180 was used to binarize the spongiosa and bone marrow in this analytical system [8]. Trabecular bone was determined by a fixed threshold. Hand-drawn contours were used to isolate the metaphyseal region of interest and trabecular compartments based on 100/250 consecutive slices. Bone micro-architecture was assessed with direct three-dimensional (3D) methods by μCT Evaluation Program (Image Processing Language v. 5.0A, Scanco). On the original 3D image, morphometric parameters were directly determined from the binarized volume of interest [19]. The parameters included: (1) the trabecular bone volume fraction (BV/TV), bone volume (BV), and total tissue volume (TV), (2) trabecular spacing (Tb.Sp.), (3) trabecular thickness (Tb.Th.), (4) the trabecular number (Tb.N.), (5) the structure model index (SMI), (6) connectivity density (Conn.D.), and (7) the bone surface to bone volume ratio (BS/BV).

### Statistical analysis

All data are presented as means ±SD. Statistical analyses were performed using the Kruskal-Wallis analysis of variance (ANOVA) test, followed by the least significant difference test (Student’s *t-*test) for multiple comparisons using SPSS software (version 13.0, SPSS, Inc., Chicago, IL, USA). A *P* value of <0.05 was considered statistically significant.

## Results

### Survival outcome

One mouse in the zoledronic acid-treated group accidently died due to anesthesia while all other mice survived without any incidence of infection.

### β-Catenin expression

**Fig 1:**
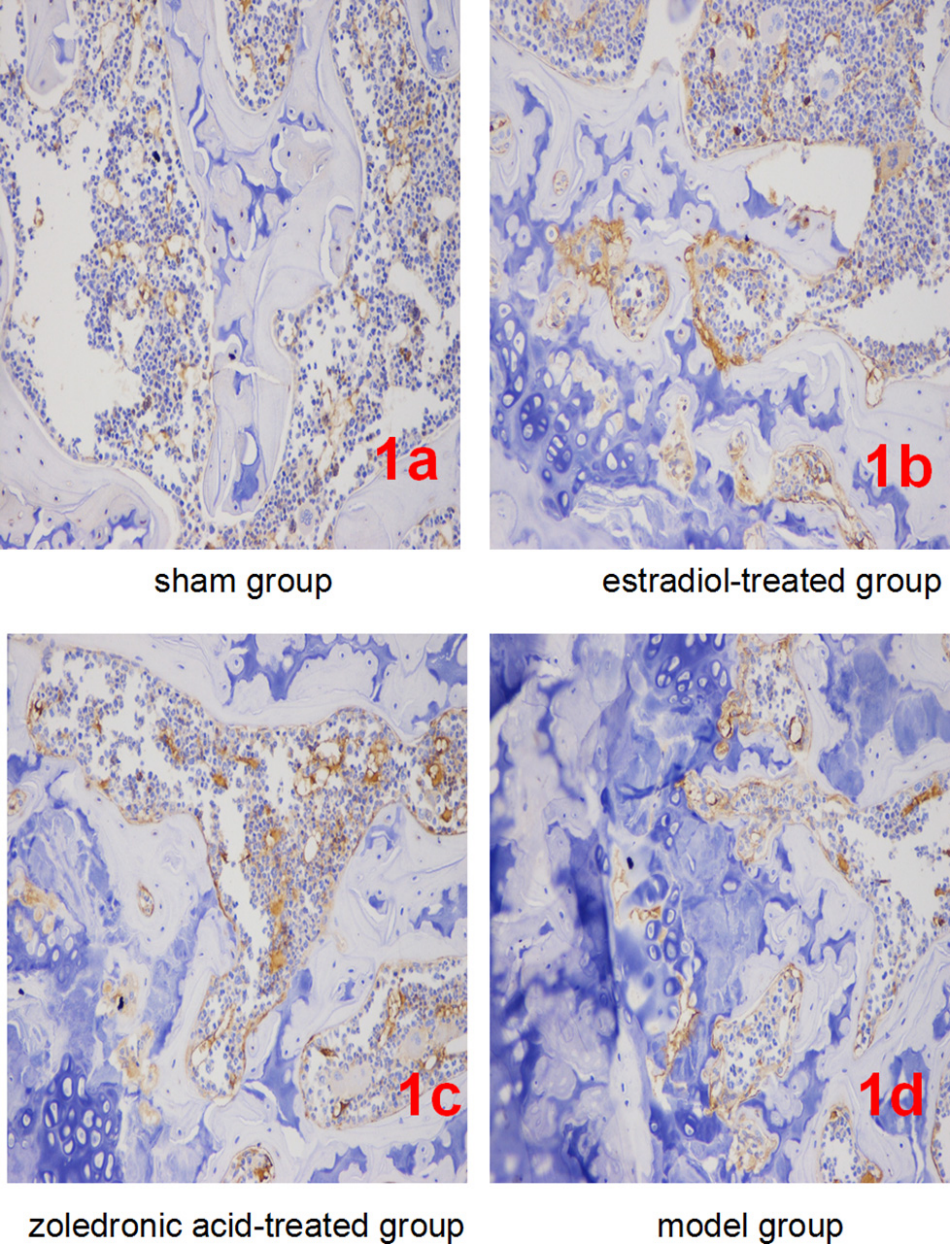
Expression of β-catenin in each group (IHC, ×400). (1a-1d) Representatives of β-catenin staining in bone tissue cells in sham/normal group (1a), ovariectomized osteoporosis associated with estradiol-treated group (1b), ovariectomized osteoporosis associated with zoledronic acid-treated group (1c), and ovariectomized osteoporosis model group (1d). All photomicrographs images at 400x magnification.

β-catenin expression per field of view was significantly lower in bone tissue cells of the ovariectomized osteoporosis model group compared with that in the sham group (*P* <0.01). In contrast, β-catenin expression in bone tissue cells of the estradiol-treated and the zoledronic acid-treated group was significantly higher than that in the ovariectomized osteoporosis model (*P* <0.01), but was significantly lower than that in the sham group (*P* <0.01). No differences in β-catenin expression were observed between bone tissue cells of the estradiol-treated and the zoledronic acid-treated group (Fig 1). Details of compared this β-Catenin expression have been described previously [20].

### Three dimensional micro-CT imaging

**Fig 2:**
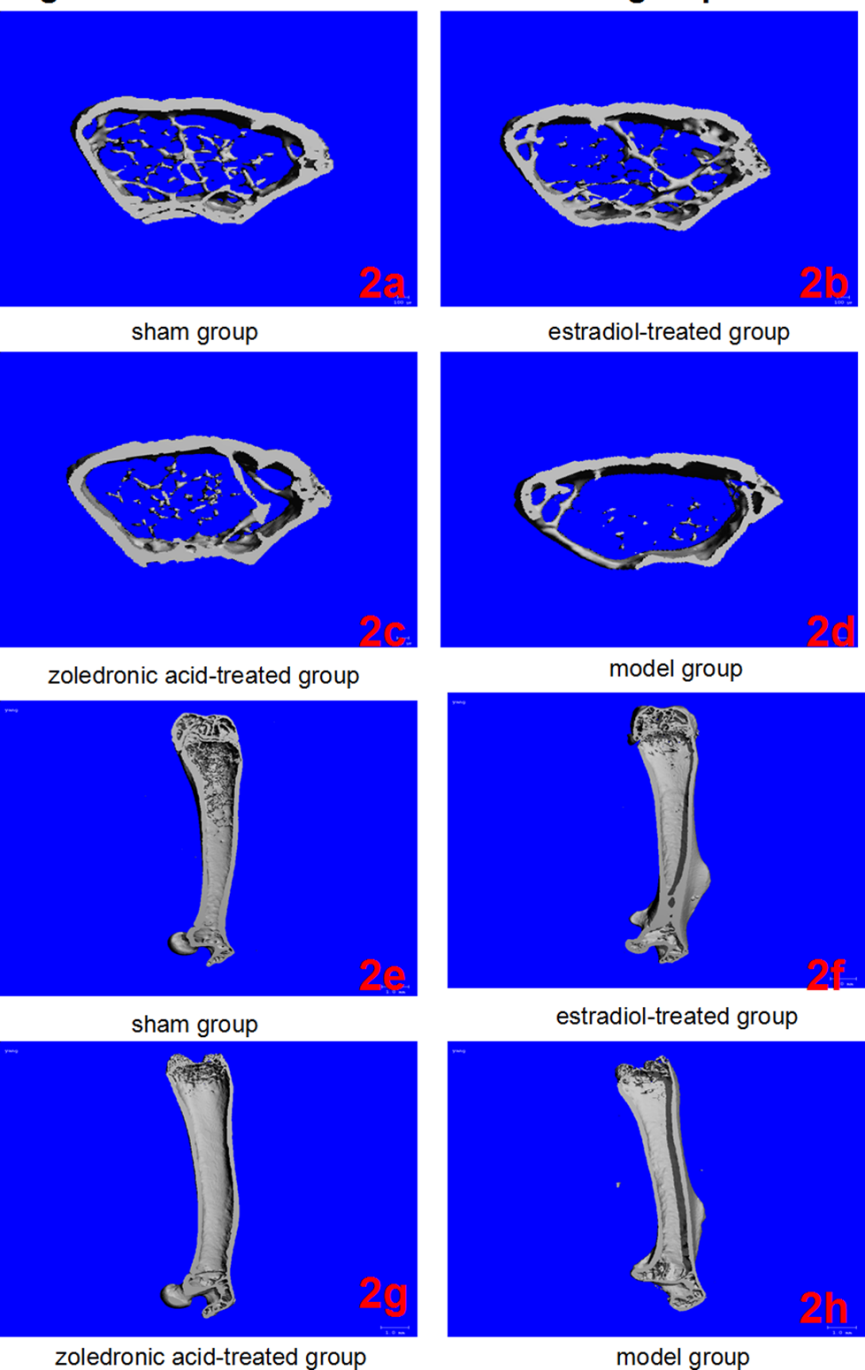
Micro-CT of left femurs in the four groups. (2a-2d) Three-dimensional images reconstructed from micro-CT analysis on the cortical and trabecular bone microarchitecture of distal femoral metaphysis (cross section) in four groups. (2e-2h) Three-dimensional images reconstructed from micro-CT analysis on the cortical and trabecular bone microarchitecture of whole left femur (longitudinal section) in four groups.

**Fig 3:**
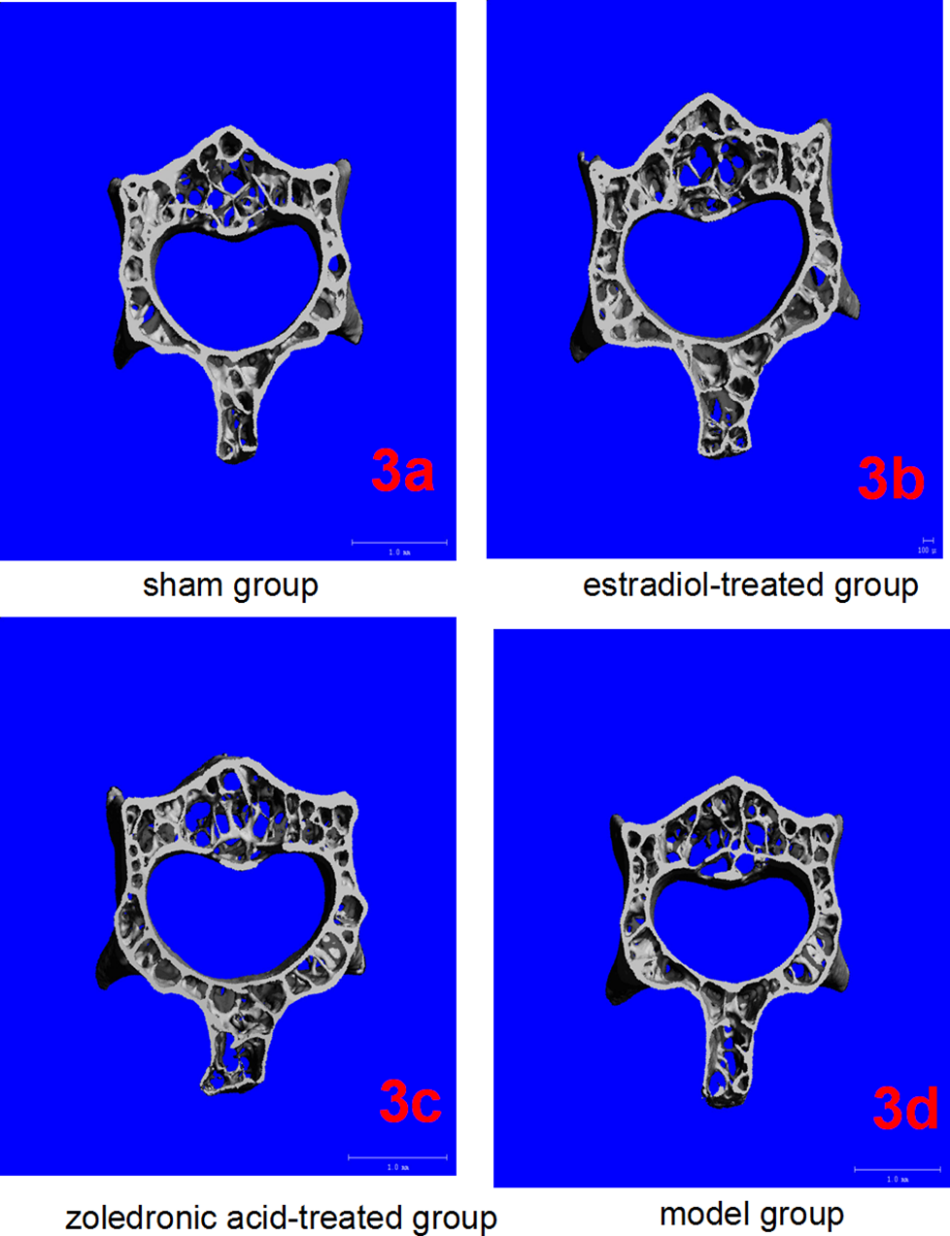
Micro-CT of lumbar vertebrae in the four groups. (3a-3d) Three-dimensional images reconstructed from micro-CT analysis on the cortical and trabecular bone microarchitecture of 3rd lumbar (cross section) in four groups.

**Fig 4:**
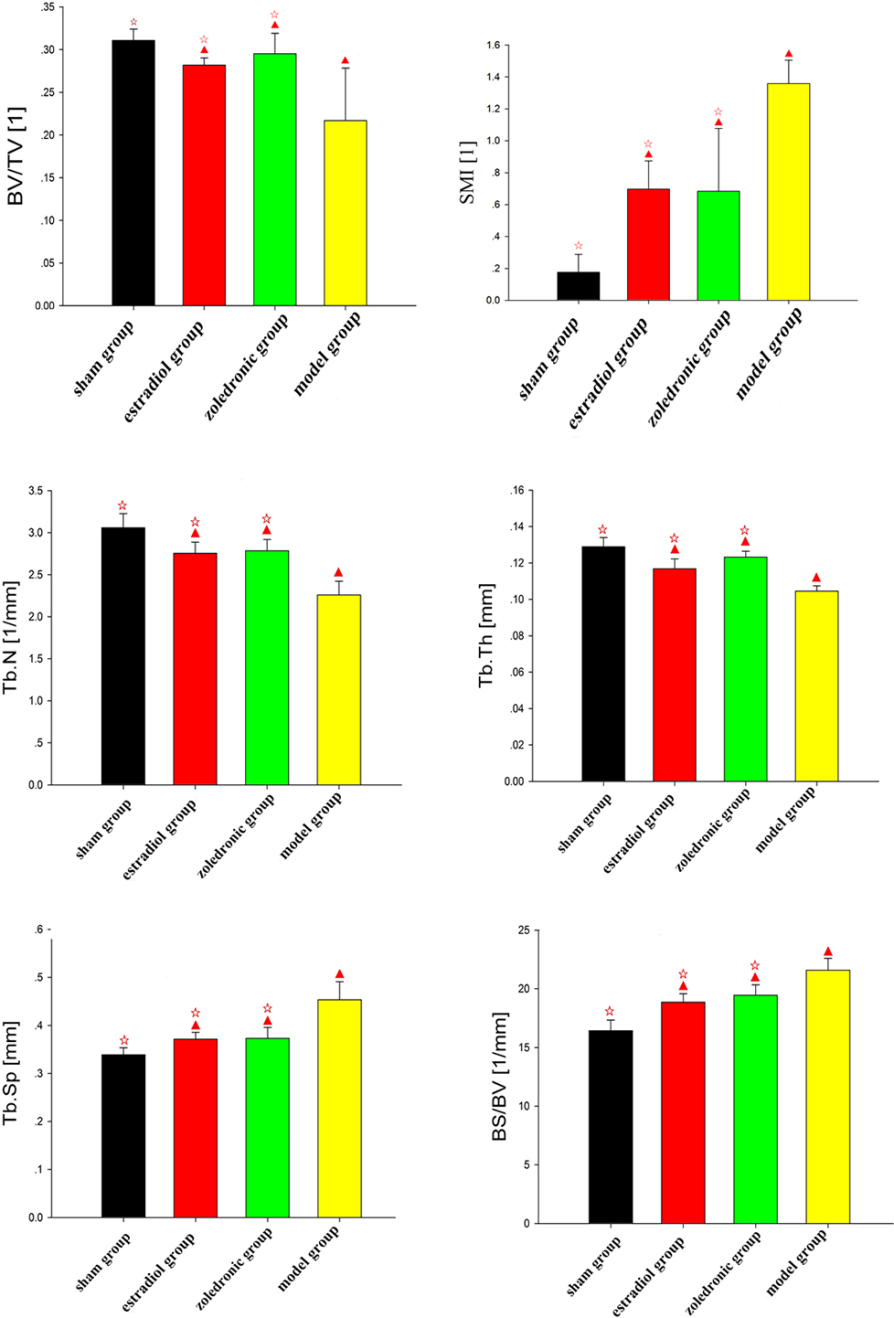
Comparison of the bone morphology parameters of femurs in the four groups. ^▲^ *p* <0.01 vs. sham group; ^☆^ *p* <0.01 vs. model group. BV, bone volume; TV, total tissue volume; BV/TV, bone volume to total tissue volume ratio (bone volume fraction); SMI, structure model index; Tb.N, trabecular number; Tb.Th, trabecular thickness; Tb.Sp, trabecular spacing; BS/BV, bone surface to bone volume ratio.

**Fig 5:**
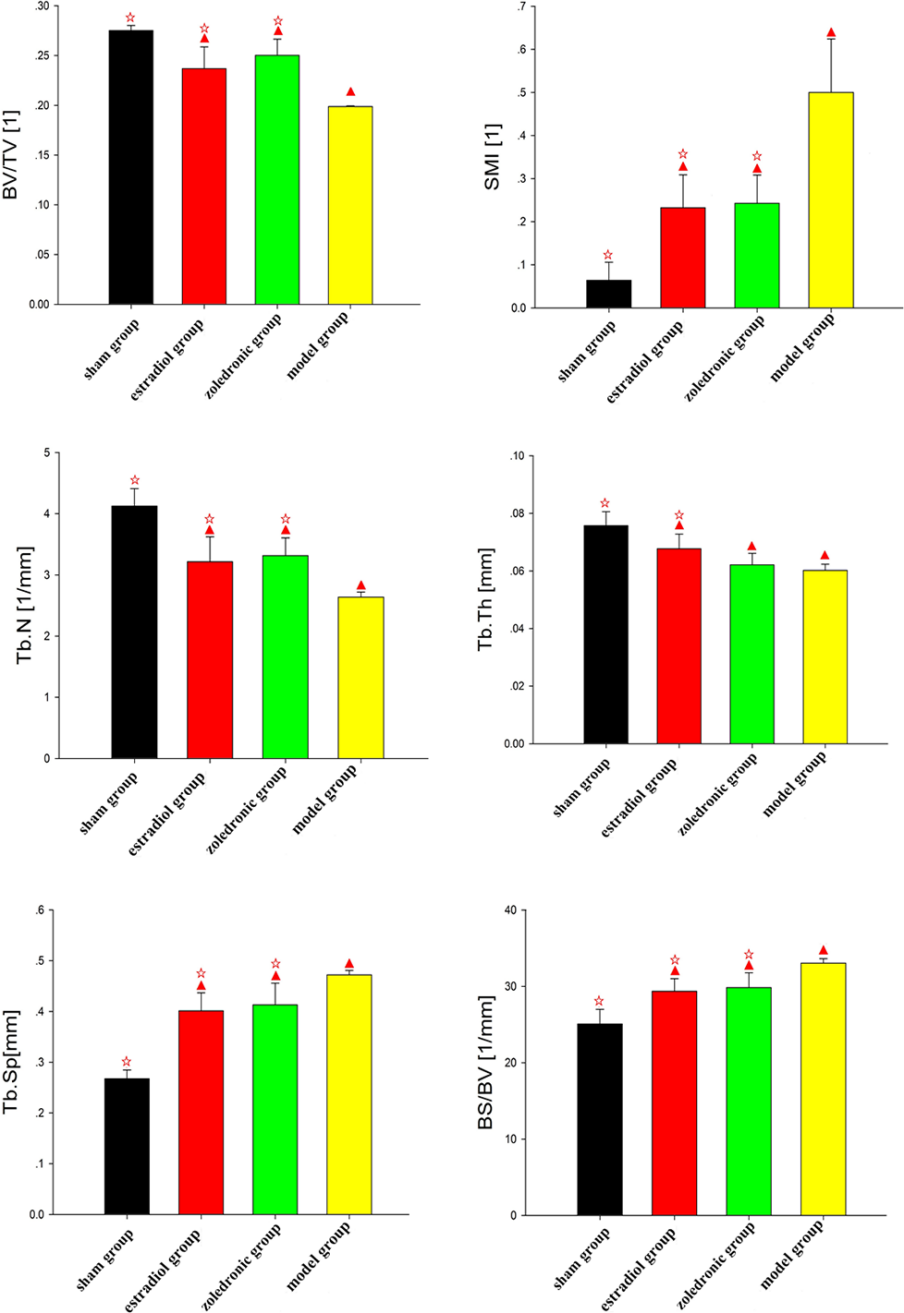
Comparison of the bone morphology parameters of lumbar vertebrae in the four groups. ^▲^ *p* <0.01 vs. sham group; ^☆^ *p* <0.01 vs. model group. BV, bone volume; TV, total tissue volume; BV/TV, bone volume to total tissue volume ratio (bone volume fraction); SMI, structure model index; Tb.N, trabecular number; Tb.Th, trabecular thickness; Tb.Sp, trabecular spacing; BS/BV, bone surface to bone volume ratio.

The parameters of the trabecular bone after 3-D reconstruction demonstrated that, in animals of the ovariectomized osteoporosis model group, the bone density was significantly attenuated, the bone micro-architecture severely impaired, the trabeculae were thin and less dense, and the connectivity was reduced, when compared with the sham groups. The rod-like trabeculae outnumbered plate-like structures, and larger inter-trabecular spaces were present in animals of the ovariectomized osteoporosis model group (Figs 2 and 3). Furthermore, their BV/TV, Tb.N., and Tb.Th. were significantly lower and their SMI, Tb.Sp., and BS/BV were significantly higher compared with the animals in the sham group (both *P* <0.01), indicating the successful establishment of the experiment model (Figs 4 and 5).

Compared with the trabecular bone structure of the ovariectomized osteoporosis model group, that of both the estradiol-treated and the zoledronic acid-treated groups was significantly better. This was shown by a higher number of trabeculae parallel-arranged in the same direction; decreased porosity; and a significantly increased BV/TV, Tb.N., and Tb.Th. compared with those of the ovariectomized osteoporosis model group (*P* <0.01). The SMI, Tb.Sp., and BS/BV, however, were significantly lower than in the ovariectomized osteoporosis model group (*P* <0.01) (Figs 4 and 5).

No significant differences were found in the micro-CT imaging parameters of the femur between animals of the estradiol-treated and the zoledronic acid-treated group. The only difference was found in the Tb.Th. of the lumbar vertebra, which was significantly higher in animals of the estradiol-treated than that of the zoledronic acid-treated group (*P* <0.01) (Figs 4 and 5).

## Discussion

The pathological changes caused by osteoporosis are both qualitative and quantitative. Quantitative effects include a decrease in BMD, while qualitative effects include changes in the growth condition of the trabecular bone, bone mineralization, area of bone accumulation, and micro-injury, resulting in regional morphological changes such as a decrease in the volume of both cortical and trabecular bones, and the disruption and thinning of trabeculae. An increasing body of evidence has demonstrated that BMD measurement is not a comprehensive method for the evaluation of changes in the mechanical properties of osteoporotic bones [21]. For example, slight changes (5–8%) in BMD can result in ~60% differentials in the mechanical properties of bone in response to mechanical loads [22]. Meanwhile, DEXA, a widely used 2-dimensional scanning technique for the measurement of bone mineral content, cannot differentiate between bone’s various soft tissue components, such as red and yellow bone marrow, muscle, and adipose tissue, which end up being considered as mineral content. This inevitably leads to inaccuracies (both positive and negative) in measuring BMD. Errors of up to 20% have been reported for DEXA, while the technique is even less accurate when measuring minerals in bones of low mass (osteoporotic bone or callus tissue), when errors of up to 30% have been recorded [23, 24]. Several animal experiments and clinical studies have shown that bone strength is not increased just by increasing BMD; on the contrary, a decrease in strength has been observed occasionally [25–27].

The emergence of the micro-CT technique has deepened our understanding of the microarchitecture of osteoporosis. Micro-CT is a non-invasive, 3-D X-ray imaging technique with a high resolution in the nanometer range [28]. The imaging principles and operating procedures of micro-CT are very similar to those of a conventional CT scanner, but its resolution is considerably higher than that of a spiral CT scanner acquiring authentic volume images [29, 30]. Micro-CT was originally designed for the detection of structural defects in ceramics and stress defects in metals, and technological advances have increased the resolution of this technique down to the nanometer range [31]. Currently, the orthopedic applications of micro-CT are for the most part the assessment of trabecular structure and mechanical analysis in cancellous bone. Micro-CT uses voxels as the unit of scan volume, providing more accurate quantitative information on mineral distribution in scanned sections and reconstructing 3-D trabecular structure at resolutions down to the micrometer level. This enables much more thoroughgoing investigation of the mineral content and microstructure of bone specimens and avoids the inaccuracies resulting from the interference of soft tissue and bone marrow characteristic of DEXA scanning [32]. Micro-CT scanning can differentiate between cortical and cancellous bone while acquiring high-resolution 3-D images [33]. In addition, this technique enables quantitative analysis of a wider range of data, enabling multi-aspect comparisons and providing tremendous convenience in the study of small animals, especially of mice in several mouse models of disease [34]. Of the microstructural parameters measured by the micro-CT technique, BV/TV, the percentage of trabecular bone volume to total volume of bone tissue, is one of the most important in revealing the microstructure of cancellous bone, in that it helps us calculate the volume of bone tissue in cancellous bone. Furthermore, Tb.Th. represents the average thickness of trabecular bone; Tb.N. is defined as the number of intersections between bone tissue and non-bone components within a defined length of specimen; and Tb.Sp. provides the average width of the trabecular bone marrow. Taken together, these parameters reveal the geometric features of trabecular bone. SMI is an additional variable for depicting the 3-D geometry of subjects. It represents the structural properties of trabecular bone, and is used for depicting the surface curvature of subject samples. SMI also helps us evaluate whether trabecular bone is rod-like or plate-like, which helps establish the ratio between plate-like and rod-like structures. Ideal plate-like and rod-like structures have an SMI value of 0 and 3, respectively. Under normal physiological conditions, cancellous bone is composed of a mixture of plate-like and rod-like structures, with an SMI value within the 0–3 range. In osteoporosis, plate-like trabeculae are converted to rod-like structures, thus increasing the SMI value.

In this study, we discovered that in ovariectomized mice the number of trabeculae was lower, the inter-trabecular space greater, and the connectivity of the trabecular bone structure was disrupted. The SMI values of ovariectomized animals were higher than those of the animals in the sham group, which is consistent with previously reported results [35]. This increase in the SMI value may have been the result of enhanced osteoclastic resorption at the bone surface caused by decreased estrogen. Osteoclastic resorption increases relative to osteoblastic bone formation, resulting in a gradual expansion of the resorption area. This develops into a condition where plate-like trabeculae are gradually converted into rod-like structures. In addition, the BV/TV, Tb.N., and Tb.Th. of the animals in the estradiol-treated group were significantly higher than in the animals of the ovariectomized osteoporosis model group (*P* <0.01); while the Tb.Sp. and BS/BV, were significantly lower than in the animals of the ovariectomized osteoporosis model group (*P* <0.01). This was partly because the post-ovariectomy decline of estrogen results in a significant alteration of the microstructure of cancellous bone, characterized by the conversion of plate-like trabeculae to a rod-like structure, increased trabecular separation, and a reduced number of trabecular bones. This has a deleterious effect on the mechanical properties of the bone. Administering estrogen is able partially to restore the microstructure of cancellous bone, thereby improving its mechanical properties. In the current study, no significant differences were observed in the micro-CT imaging data of the lumbar vertebra and femur of animals in the estradiol-treated and the zoledronic acid-treated group.

Wnt/β-catenin signaling is currently recognized as an important regulator of bone mass and bone cell differentiation. It interacts with other signaling pathways, including estrogen receptor signaling [36]. Both the Wnt/β-catenin and estrogen receptor signaling pathways play an important role in bone remodeling, particularly in menopausal women [37]. The main purpose of this study was to investigate the mechanisms by which the phytoestrogen function of zoledronic acid prevents bone loss in estrogen deficiency, with a specific emphasis on the activation of β-catenin expression. The present study shows that β-catenin expression was not different between the estradiol-treated and the zoledronic acid-treated group, but that its expression was significantly higher in both groups than in the ovariectomized osteoporosis model group. Our preliminary studies showed a significant positive correlation between the serum levels of β-catenin and those of osteoprotegerin (OPG), a negative correlation with sclerostin, and also negative correlation with the level of receptor activator for the nuclear factor kappa B ligand (RANKL)/OPG [38, 39], all of which suggest that β-catenin signaling contributes to bone growth and bone remodeling. These findings are consistent with our micro-CT imaging results. Taken together, our results strongly suggest that zoledronic acid strengthens the quality and quantity of bone through the activation of β-catenin expression, which is mediated through a non-genomic estrogenic action. To our knowledge, our study reporting on the action of zoledronic acid on the β-catenin expression pathway is in accord with Tai TW [16] results.

A previous study by the authors demonstrated that a low level of β-catenin is likely to be associated with the onset of post-menopausal osteoporosis. The interaction between the Wnt/β-catenin signaling pathway and the RANKL/RANK/OPG signaling pathway might thus play an important role in the pathogenesis of post-menopausal osteoporosis [38, 39]. Zoledronic acid is a potent anti-resorptive agent which has been approved for the treatment of various disorders characterized by increased osteoclast-mediated bone resorption, such as estrogen depletion, aging, and disuse [40]. Zoledronic acid was effective on osteoclastic activity, such as preventing osteoclast formation, reducing osteoclast-mediated bone resorption activities, and inducing osteoclast apoptotic cell death [40, 41]. In this experiment, the BV/TV, Tb.N., Tb.Th. and BS/BV of animals in the zoledronic acid treated group were significantly higher than in the model group (*P* <0.01); conversely, the SMI, Tb.Sp. and BS/BV were significantly lower than in the model group (*P* <0.01), suggesting an increase in the micro-quality of the bone, including the shape and structure as well as the 3-D bone geometry of the trabeculae. Consistent with previous findings, the results of this present study further revealed the positive therapeutic effect of zoledronic acid in the treatment of osteoporosis, and showed that this could be the result of its action in elevating the bone mass through improved expression of β-catenin [42, 43]. We also found that it resulted in significant improvement in the trabecular microstructure, an increase in the quantity of parallel-arranged trabecular bone structures, and a reduction in porosity [44–46]. However, further study is required to elucidate the mechanism in more detail.

## Supporting Information

S1 Dataset The data set and supporting information for the bone morphology parameters of femur. The values of the bone morphology parameters of micro-CT in mice femur are indicated.
(XLS)

S2 Dataset The data set and supporting information for the bone morphology parameters of lumbar vertebrae. The values of the bone morphology parameters of micro-CT in mice lumbar vertebrae are indicated.
(XLS)
